# Recurrent delirium over 12 months predicts dementia: results of the Delirium and Cognitive Impact in Dementia (DECIDE) study

**DOI:** 10.1093/ageing/afaa244

**Published:** 2020-12-16

**Authors:** Sarah J Richardson, Daniel H J Davis, Blossom C M Stephan, Louise Robinson, Carol Brayne, Linda E Barnes, John-Paul Taylor, Stuart G Parker, Louise M Allan

**Affiliations:** Translational and Clinical Research Institute, Faculty of Medical Sciences, Newcastle University, Newcastle upon Tyne NE4 5PL, UK; MRC Unit for Lifelong Health and Ageing at UCL, London WC1E 7HB, UK; Institute of Mental Health, Division of Psychiatry and Applied Psychology, School of Medicine, University of Nottingham, Nottingham NG7 2TU, UK; Institute of Population Health Sciences, Faculty of Medical Sciences, Newcastle University, Biomedical Research Building, Newcastle upon Tyne NE4 5PL, UK; Cambridge Public Health, Cambridge Institute of Public Health, University of Cambridge, Cambridge CB2 0SR, UK; Cambridge Public Health, Cambridge Institute of Public Health, University of Cambridge, Cambridge CB2 0SR, UK; Translational and Clinical Research Institute, Faculty of Medical Sciences, Newcastle University, Newcastle upon Tyne NE4 5PL, UK; Institute of Population Health Sciences, Faculty of Medical Sciences, Newcastle University, Biomedical Research Building, Newcastle upon Tyne NE4 5PL, UK; College of Medicine and Health, South Cloisters, University of Exeter, Exeter EX1 2LU, UK

**Keywords:** delirium, dementia, epidemiology, cohort study, older people

## Abstract

**Background:**

Delirium is common, distressing and associated with poor outcomes. Previous studies investigating the impact of delirium on cognitive outcomes have been limited by incomplete ascertainment of baseline cognition or lack of prospective delirium assessments. This study quantified the association between delirium and cognitive function over time by prospectively ascertaining delirium in a cohort aged ≥ 65 years in whom baseline cognition had previously been established.

**Methods:**

For 12 months, we assessed participants from the Cognitive Function and Ageing Study II-Newcastle for delirium daily during hospital admissions. At 1-year, we assessed cognitive decline and dementia in those with and without delirium. We evaluated the effect of delirium (including its duration and number of episodes) on cognitive function over time, independently of baseline cognition and illness severity.

**Results:**

Eighty two of 205 participants recruited developed delirium in hospital (40%). One-year outcome data were available for 173 participants: 18 had a new dementia diagnosis, 38 had died. Delirium was associated with cognitive decline (−1.8 Mini-Mental State Examination points [95% CI –3.5 to –0.2]) and an increased risk of new dementia diagnosis at follow up (OR 8.8 [95% CI 1.9–41.4]). More than one episode and more days with delirium (>5 days) were associated with worse cognitive outcomes.

**Conclusions:**

Delirium increases risk of future cognitive decline and dementia, independent of illness severity and baseline cognition, with more episodes associated with worse cognitive outcomes. Given that delirium has been shown to be preventable in some cases, we propose that delirium is a potentially modifiable risk factor for dementia.

## Key points

- Delirium increases risk of future cognitive decline.
- Longer duration, more severe delirium and more episodes of delirium were associated with worse cognitive outcomes.
- Delirium may be a potentially modifiable risk factor for dementia.

## Background

Delirium is a sudden onset and fluctuating cognitive disorder, frequently precipitated by acute illness, which specifically affects attention and level of arousal. Delirium is highly distressing and common, affecting 1 in 4 older people in hospital [1,2]. Delirium is independently associated with increased institutionalisation and mortality, longer lengths of hospital stay and more hospital-acquired complications, culminating in substantial additional healthcare costs [2–4]. Delirium is a strong predictor of new-onset dementia as well as acceleration of existing cognitive decline [5–7].

The degree to which delirium is associated with permanent changes in cognition is unclear because most studies (outside specific elective surgical settings) have not assessed prior cognitive function [8–10]. In contrast, the studies that have ascertained prior cognitive function lack reliable delirium measures [11–13]. Therefore, the degree to which baseline cognition and delirium separately or together contribute to cognitive outcomes remains unknown. A further mediator may be acute hospitalisation itself. This has been shown to adversely affect trajectories of cognitive decline, even when delirium has not been specifically ascertained [14–16]. This implies that delirium and/or its acute causes can contribute to the overall burden of dementia.

In order to address the relative contribution of acute and chronic factors in determining subsequent cognitive impairment, the Delirium and Cognitive Impact in Dementia (DECIDE) study aimed to measure the effect of delirium on cognition, independent of illness severity, in a population-based cohort study of incident dementia. A further objective was to quantify the association of other features of delirium, including number of episodes and duration, on cognitive outcomes.

## Methods

### Population

The DECIDE study is a prospective sample nested within the Cognitive Function and Ageing Study II (CFAS-II) [17,18]. CFAS-II is a large, population-based cohort from three geographical areas in the UK, including Newcastle upon Tyne, measuring prevalence and incidence of dementia [18]. At baseline (2011–2013), 1,751 participants aged ≥ 65 years were recruited to CFAS II-Newcastle sample. Global as well as domain-specific cognitive function was assessed using the Geriatric Mental State, the Cambridge Cognitive Examination and the Mini-Mental State Examination (MMSE). An algorithmic approach to the diagnosis of dementia, depression and anxiety was made using the Automated Geriatric Examination for Computer Assisted Taxonomy, drawing on respondent and observer ratings [18]. The full content of the interviews is available online (http://www.cfas.ac.uk/). All members of CFAS II-Newcastle live within the catchment area of the Newcastle Hospitals NHS Foundation Trust.

Surviving members of CFAS II-Newcastle were contacted by CFAS prior to the start of the DECIDE study. Participants were provided with written information and given the opportunity to opt-out of further contact. All non-objecting members of CFAS-II Newcastle, with or without dementia, were eligible to participate in DECIDE if admitted to the Royal Victoria Infirmary or Freeman Hospital as an emergency or electively between 5 January 2016 and 5 January 2017. We were alerted to admissions by a Recurring Admission Patient Alert on the electronic records system. If the participant themselves lacked capacity, an appropriate personal consultee was requested to provide written confirmation of willingness to participate. Once recruited, participants were seen on each subsequent hospital admission during the study period. Participants were excluded if: they lacked capacity to consent and we were unable to identify or contact an appropriate personal consultee, they were receiving end of life care, they were being isolated for infection control reasons, they were expected to be in hospital for fewer than 24 h.

### Exposures

#### Delirium

Participants were assessed daily throughout their admission. Delirium was ascertained using a standardised procedure based on DSM-5 criteria, combining objective testing of the participant, and information gained from informants (usually nurses, next of kin or clinical records), with structured observations made by two assessors [19]. *Disturbance in attention* was evaluated using months of the year backwards and digit span, and arousal was recorded using the Observational Scale of Level of Arousal and the modified Richmond Agitation and Sedation Scale [20–22]. *Disturbance in cognition* was evaluated using 3 item recall, 10 orientation questions, 3 stage commands and any evidence of perceptual disturbances along with observations by the examiner during the interview. *Acute onset and fluctuating course*, change from baseline and *evidence of underlying medical condition* were obtained from informant history from nursing staff, next of kin and clinical records. In order to operationalise grades of delirium exposure, we considered three related quantities: (1) number of episodes; (2) total days with delirium; (3) peak delirium severity scores recorded using the Memorial Delirium Assessment Scale (MDAS) [22].

As far as possible, we assessed participants daily during their hospital admission(s) for the presence of delirium. If it was not possible to review participants prospectively at any particular time point, due to illness, refusal or study capacity, we used a validated tool to retrospectively review the medical records for a diagnosis of delirium [23]. In those participants in whom a diagnosis of delirium was not possible prospectively or was uncertain, study information was sent to an expert consensus panel [LMA, DHJD, SGP]. The vignettes contained a complete copy of the data collection forms for the participant along with all information collected retrospectively from the medical records using the validated tool [23]. Two members of the expert panel [LMA, DHJD] independently reviewed each case and if they disagreed, a third member [SGP] reviewed the case and a majority decision was applied. The panels were tasked with determining whether delirium was present or absent.

#### Illness severity

Concurrent illness severity was recorded using APACHE II [24].

#### Other variables

We collected baseline data on recruitment to DECIDE including age, sex, comorbidity (recorded using the Cumulative Illness Rating Scale for Geriatrics (CIRS-G)) and frailty (recorded using the Clinical Frailty Scale) [25,26].

### Outcomes

We invited all participants recruited in hospital, with and without delirium, for follow-up 12 months after their most recent hospital discharge. This consisted of a home visit to complete the CFAS II interview for dementia ascertainment [18]. The primary outcomes were dementia status and MMSE score at 12 months after hospital discharge in comparison to baseline cognitive function from CFAS II.

### Data collection

All data collection was carried out by the chief investigator [SJR] and a part-time research nurse. At monthly intervals throughout the study, we completed joint assessments of a sample of participants to optimise consistency between assessors. Training to deliver the standardised computerised interview at 1-year follow up was provided by the CFAS team based at University of Cambridge. They also had oversight of on-going quality control, using audio recording of interviews with consenting participants.

### Statistical analysis

We evaluated between groups differences using an independent *t*-test, Kruskall–Wallis test or chi-squared test depending on data type and distribution. We used multiple regression to determine whether delirium was associated with cognitive decline or dementia, independent of baseline cognition and illness severity along with other relevant confounders including age, education, sex, frailty, baseline cognition, time between baseline and follow up interviews and comorbidities. We used multiple regressions to estimate the effect of peak delirium severity, delirium duration and total number of episodes of delirium. We investigated patterns of missing data and delirium cases evaluated by the expert panel in sensitivity analyses. All statistical analyses were performed on STATA Version 15.

## Results

### Characteristics

**Figure 1:**
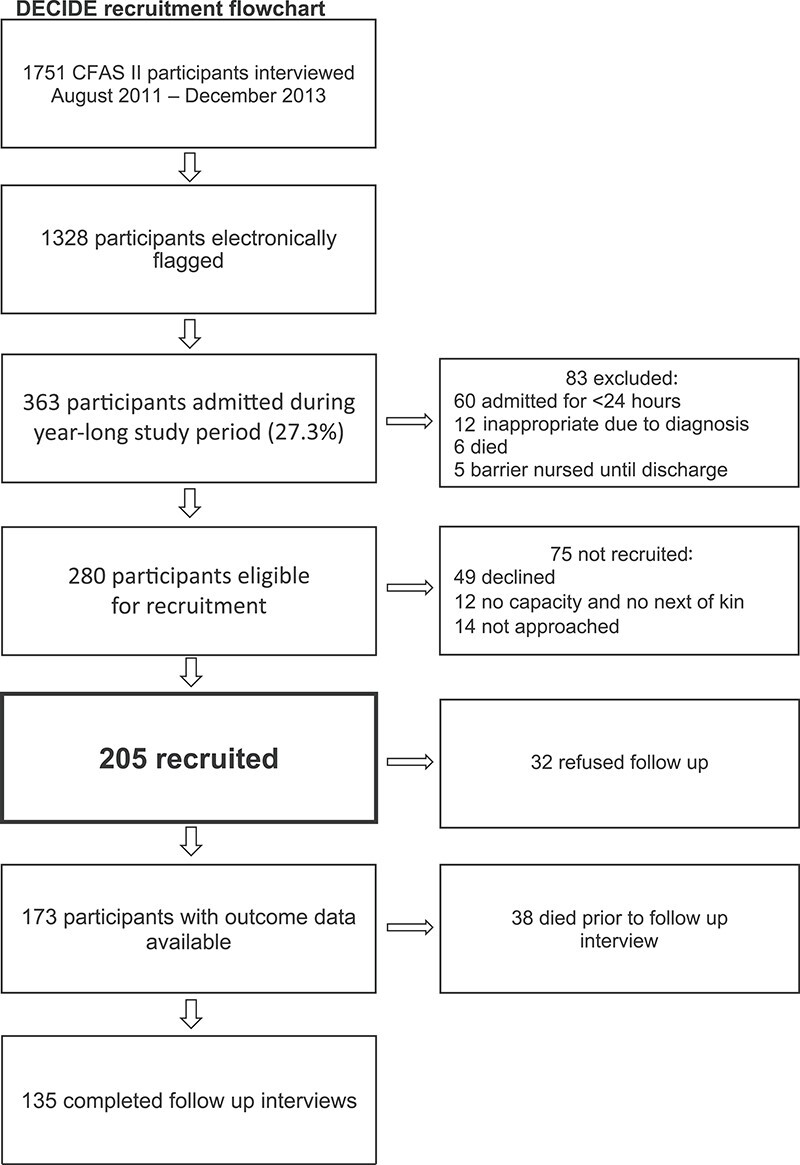
Flowchart showing recruitment to the DECIDE study along with the proportion of participants who completed follow up interviews at 1 year, along with the reasons for non-completion at each stage.

Of the 1,751 CFAS II-Newcastle participants with baseline data, 1,328 were surviving and non-objecting at the start of the DECIDE study. During the year of recruitment, 363 (27·3%) were admitted to hospital, 280 were eligible for DECIDE and 205 were recruited (73·2%) (Figure 1). There were no significant differences in baseline characteristics between those who were recruited to DECIDE and those who were not (Supplementary Table 1). Baseline characteristics of DECIDE participants are summarised in Table 1. Ninety six of the 205 participants were readmitted during the study period (46·8%), with a total of 186 readmissions. Median length of stay for all admissions was 5 days (IQR 10·3 days).

**Table 1:** Baseline characteristics of participants

Variable	Total (*n* = 205)	Delirium (*n* = 82)	No delirium (*n* = 123)	*P* value
Age (mean, SD)	82.0 ± 6.5	84.7 ± 6.5	80.3 ± 5.9	*P* < 0.001
Sex (% women)	53.2	48.8	56.1	*P* = 0.304
≤10 years in full time education (%)	71.2	78.1	66.7	*P* = 0.078
Baseline cognition (MMSE) (mean, SD)	26.3 ± 3.2	24.9 ± 3.7	27.3 ± 2.5	*P* < 0.001
% living in 24 h care	5.9	7.3	4.9	*P* = 0.466
Comorbidity score (mean, SD)	8.6 ± 4.3	10.1 ± 4.2	7.6 ± 4.1	*P* < 0.001
Clinical frailty score (mean, SD)	4.3 ± 1.4	4.9 ± 1.2	3.8 ± 1.4	*P* < 0.001

Demographic data for all DECIDE participants, those who developed delirium during the study period and those who did not develop delirium during this time.

Age, accommodation, comorbidity score (total CIRS-G score) and frailty (total Clinical Frailty Scale score) recorded on recruitment to DECIDE.

**Table 2:** Delirium as an independent predictor of new dementia diagnosis

	Analysis 1: Delirium during 2016 (yes)	Analysis 2: Total number of days with delirium during the year-long study period		Analysis 3: Total number of episodes of delirium during the year-long study period		Analysis 4: Delirium severity according to peak MDAS score during the year-long study period (per point)
		1–5 days	>5 days	1 episode	>1 episode	
Odds ratio (95% confidence interval), *P* value	8.8 (1.9–41.4), 0.006	9.3 (2.0–44.2), 0.005	8.4 (0.8–85.0), 0.072	8.6 (1.8–41.1), 0.007	13.9 (1.3–151.0), 0.031	1.3 (1.1–1.5), 0.012

Results of consecutive regression analyses exploring delirium variables which independently predict new dementia diagnosis at 1 year after hospital admission (*n* = 135). Other variables not shown but adjusted for in regression analysis were: age (at recruitment to DECIDE), sex, education, illness severity (peak total APACHE II score), baseline cognition (MMSE score at baseline), co-morbidity (total CIRS-G score recorded on recruitment to DECIDE), frailty (total Clinical Frailty Score recorded on recruitment to DECIDE, included as a continuous variable) and time between baseline and follow-up interviews.

**Table 3:** Delirium as an independent predictor of MMSE score at follow-up

	Analysis 1: Delirium during 2016 (yes)	Analysis 2: Total number of days with delirium during the year-long study period		Analysis 3: Total number of episodes of delirium during the year-long study period		Analysis 4: Delirium severity according to peak MDAS score during the year-long study period (per point)
		1–5 days	>5 days	1 episode	>1 episode	
Coefficient (95% confidence interval), *P* value	−1.8 (−3.5—−0.2), 0.030	−1.7 (−3.4—−0.1), 0.044	−5.1 (−8.1—−2.1), 0.001	−1.9 (−3.6—−0.2), 0.031	−1.5 (−4.7–1.7), 0.362	−0.4 (−0.6—−0.2), 0.001

Results of consecutive regression analyses exploring delirium variables which independently predict MMSE score at 1 year after hospital admission (*n* = 135). Other variables not shown but adjusted for in regression analysis were: age (at recruitment to DECIDE), sex, education, illness severity (peak total APACHE II score), baseline cognition (MMSE score at baseline), co-morbidity (total CIRS-G score recorded on recruitment to DECIDE), frailty (total Clinical Frailty Score recorded on recruitment to DECIDE, included as a continuous variable) and time between baseline and follow-up interviews.

### Delirium

A total of 82 participants developed delirium in hospital during the study period (40%) and 24 participants had more than one episode of delirium (29%). For those who experienced delirium, the median total number of days with delirium during the study period was 3 days (IQR 6 days). Older age (OR 1·09 per year [95% CI 1·03–1·16]) and lower baseline cognition (OR 0.95 per MMSE point [95% CI 0·91–0.99]) were independently associated with delirium.

### Follow-up interviews at 12 months

One hundred and thirty five of 205 participants completed follow up interviews 1 year after hospital admission (65·9%), with 18 participants receiving a new diagnosis of dementia. A total of 38 participants had died prior to follow up (18·5%) and 32 refused follow up (15·6%) (Figure 1). Mean time between participants’ baseline interview, as part of CFAS II-Newcastle, and their follow up interview for DECIDE, was 4.5 years (SD 0.8 years).

### The association between delirium and new dementia

An episode of delirium was associated with a markedly increased risk of incident dementia (OR 8.8 [95% CI 1.9–41.4]), independent of illness severity and baseline cognition, as well as age, sex, education, comorbidity, time between interviews and frailty (Table 2). Delirium also independently predicted a decline in MMSE score at follow up interview (β −1.8 points [95% CI –3.5 to –0.2]) (Table 3). Greater exposure to delirium, in terms of number of episodes, duration and severity, was associated with worse cognitive outcomes, with greater risk of incident dementia associated with more than 1 episode of delirium (OR 13.9 [95% CI 1.3–151.0]) compared to a single episode (OR 8.6 [95% CI 1.8–41.1]) (Table 2). Additionally, having delirium for more than 5 days during the study period was independently associated with lower MMSE scores at follow up interview (β −5.1 points [95% CI –8.1 to 2.1]) compared to 1–5 days with delirium (β −1.7 points [−3.4–0.1]) (Table 3). More severe delirium was associated with lower MMSE scores at follow up interview (β −0.4 points [95% CI –0.6 to –0.2]) and incident dementia (OR 1.3 [95% CI 1.1–1.5]).

Sensitivity analyses showed that excluding the four delirium cases ascertained via consensus panel requiring a third assessor did not alter the overall results.

## Discussion

In this prospective, population-based cohort study, we have shown for the first time that delirium is associated with a new diagnosis of dementia and cognitive decline, independent of baseline cognition and illness severity. Further, repeated episodes of delirium, more days with delirium and greater severity of delirium are associated with worse cognitive outcomes. Taken together, these findings suggest that increasing grades of delirium exposure (in terms of recurrent episodes, greater severity or longer duration) confer increased risk of dementia.

A strength of DECIDE was the prospective delirium assessments using a standardised approach, including a consensus panel for borderline cases. However, not all participants were seen every day, which might not have captured all days with delirium, particularity given the fluctuating nature of the condition. We mitigated this using review of notes as part of the assessment [23]. Nesting DECIDE within an existing, well-characterised cohort with known baseline cognition was also a strength because baseline cognition could be robustly accounted for when quantifying cognitive outcomes after delirium. A limitation was the variation in time that had elapsed between baseline and follow up cognitive assessments. We included a term for this as a potential confounder in our models but it is possible that cognitive function may have deteriorated during this time.

The magnitude and direction of these independent associations are similar to those found in previous studies in which delirium ascertainment was retrospective. The first used data from the original Cognitive Function and Ageing Study cohort (CFAS I) [13], and the second used data from a population-based study of over 85-year olds in Finland [11]. We have not only confirmed the associations, but by accounting for illness severity and baseline cognition and ascertaining delirium prospectively, our results add more detail on the interrelationship between delirium and cognitive outcomes. Additionally, the relationship demonstrated between increasing delirium exposure and cognitive decline supports a dose–response relationship and strengthens evidence for a causal hypothesis [7].

There are substantial clinical implications of this association between grades of delirium exposure and incident dementia and cognitive decline because delirium itself is modifiable, and in some cases preventable [27]. This paves the way for future dementia prevention trials that focus on delirium intervention in order to robustly assess whether delirium is a modifiable risk factor for dementia. The DECIDE study serves to emphasise that delirium may have consequences well beyond the acute phase and may itself be contributing to the development and burden of dementia, significant for both individuals and populations.

## Supplementary Material

aa-20-0850-File002_afaa244
