# Supplementary material for: Recurrent delirium over 12 months predicts dementia: results of the Delirium and Cognitive Impact in Dementia (DECIDE) study
Source: Age Ageing. 2020 Dec 16;50(3):914–20. doi: 10.1093/ageing/afaa244 (PMC8099011; doi:10.1093/ageing/afaa244)
Supplement: aa-20-0850-File002_afaa244 [file aa-20-0850-file002_afaa244.docx]

Recurrent delirium over 12 months predicts dementia: results of the Delirium and Cognitive Impact in Dementia (DECIDE) study

**Supplementary material**

**Baseline characteristics of CFAS II-Newcastle participants admitted to hospital during the study period**

| Variable | Admitted but not recruited (n=158) | Recruited to DECIDE (n=205) | P value |
| --- | --- | --- | --- |
| Age (mean, SD) | 78.1 +/- 6.5 | 78.6 +/- 6.5 | p=0.457 |
| Sex (% women) | 58.2 | 53.2 | p=0.337 |
| ≤10 years in full time education (%) | 73.9 | 71.2 | p=0.574 |
| Baseline cognition (MMSE) (mean, SD) | 25.6 +/- 5.1 | 26.3 +/- 3.2 | p=0.089 |

Supplementary table 1: Demographic data for all CFAS II-Newcastle participants admitted to hospital during the study, comparing those who were recruited to DECIDE with those who were not.

All variables were recorded at baseline interview by CFAS II.
